# Use of Lipid-Modifying Agents for the Treatment of Glomerular Diseases

**DOI:** 10.3390/jpm11080820

**Published:** 2021-08-21

**Authors:** Mengyuan Ge, Sandra Merscher, Alessia Fornoni

**Affiliations:** Peggy and Harold Katz Family Drug Discovery Center, Katz Family Division of Nephrology and Hypertension, Department of Medicine, Miller School of Medicine, University of Miami, Miami, FL 33136, USA; mengyuange@med.miami.edu (M.G.); smerscher@med.miami.edu (S.M.)

**Keywords:** lipids, podocytes, glomerular diseases, therapies, proteinuria, nephrotic syndrome

## Abstract

Although dyslipidemia is associated with chronic kidney disease (CKD), it is more common in nephrotic syndrome (NS), and guidelines for the management of hyperlipidemia in NS are largely opinion-based. In addition to the role of circulating lipids, an increasing number of studies suggest that intrarenal lipids contribute to the progression of glomerular diseases, indicating that proteinuric kidney diseases may be a form of “fatty kidney disease” and that reducing intracellular lipids could represent a new therapeutic approach to slow the progression of CKD. In this review, we summarize recent progress made in the utilization of lipid-modifying agents to lower renal parenchymal lipid accumulation and to prevent or reduce kidney injury. The agents mentioned in this review are categorized according to their specific targets, but they may also regulate other lipid-relevant pathways.

## 1. Introduction

Lipids are important constituents of cell membranes and play a pivotal role in energy production. However, lipid accumulation in non-adipose tissue can cause a series of problems, ultimately leading to organ failure. For instance, when the lipid content in the heart exceeds physiological limits, individuals are at risk of developing cardiomyopathy, eventually resulting in heart failure [1,2]. An increase in lipids in the liver can cause non-alcoholic fatty liver diseases ranging from fatty liver to non-alcoholic steatohepatitis (NASH) [3,4]. Evidence also suggests that lipid accumulation in pancreatic islets is associated with insulin resistance [5]. Fat accumulation in the kidney was recognized a long time ago [6,7]; however, whether this fatty infiltration was the cause or the consequence of kidney disease was never established. More recently, clinical and experimental studies have suggested that lipotoxicity may have a major causative role in the progression of chronic kidney diseases (CKD) [8].

In humans, the total lipid content in the kidney is approximately 3% of the kidney wet weight. While phospholipids account for more than half of the lipid composition of renal cells, triglycerides, free fatty acids and cholesterol esters constitute the majority of neutral lipids [9]. Podocytes, which are terminally differentiated cells of the kidney filtration barrier, are most susceptible to damage caused by lipid overload [10]. These findings initiated a major drug-development effort to develop new drugs or to repurpose existing drugs aimed at reducing the lipid surplus in non-adipose tissue. In this review, we will focus on the recent progress made in the use of lipid-modifying agents to lower intracellular lipids in glomerular diseases. We will provide an insight into the potential use of these agents as therapeutic therapies. Of the many lipid-relevant pathways of interest, we will focus our attention on cholesterol and fatty acid metabolism as we recently reviewed the role of sphingolipids in the progression of kidney diseases elsewhere [11].

## 2. Hereditary Diseases Support a Role of Lipid Dysmetabolism in the Development of Nephrotic Syndrome (NS)

The lack of proteinuria and renal failure in patients with familial hyperlipidemia [12] suggests a dissociation between systemic and renal lipids in the pathogenesis of kidney diseases and indicates that cholesterol uptake from the circulation to the renal parenchyma may not be a primary contributor to the progression of kidney diseases. Additionally, patients with a gain-of-function mutation in the hydroxy-3-methylglutaryl-CoA reductase (HMGCR) gene, which codes for the rate-limiting enzyme of the cholesterol synthesis pathway, do not develop kidney disease [13]. This is consistent with the fact that while the use of statins is recommended in all patients with CKD to target their intrinsic cardiovascular morbidity and mortality, statins do not halt the progression of kidney diseases [14]. On the contrary, several genetic diseases with impairment of cholesterol efflux and esterification can present with proteinuria, NS, and foamy podocytes in kidney biopsies, strongly supporting a causative role of impaired cholesterol efflux in the pathogenesis of proteinuric kidney diseases. Similarly, risk allele variants of genes involved in cholesterol efflux are associated with kidney disease [15].

### 2.1. Genetic Disorders Associated with Genes Involved in Reverse Cholesterol Transport

In several genetic disorders with mutations in genes that are important for proper lipid homeostasis, patients present with renal injury. For instance, Tangier disease is a rare autosomal recessive disorder associated with mutations in the ATP-binding cassette subfamily A member 1 (ABCA1) [16], and patients with the disease present with a decrease in high-density lipoproteins (HDL), accumulation of cholesterol esters in multiple organs, mild proteinuria and foamy podocytes in kidney biopsies [17]. Additionally, missense mutation of the LIM homeobox transcription factor 1 beta (LMX1β) causes nail-patella-like renal disease. This mutation is associated with reduced ABCA1 and ABCG1 expression and increased lipid droplet accumulation in podocytes, thus leading to podocyte injury [18]. Moreover, deficiency of lecithin-cholesterol acyltransferase (LCAT), the enzyme that converts cholesterol to cholesteryl esters on the surface of HDL [19], which is caused by mutations in the LCAT gene, is responsible for some cases of nephrotic syndrome [20,21]. LCAT deficiency is also associated with abnormalities of HDL maturation and reduction in HDL-mediated reverse cholesterol transport, resulting in glomerular lipid deposition with the accumulation of foam cells [20,22].

### 2.2. Apolipoprotein L1 (APOL1)-Associated Nephropathy

Patients carrying genetic variants of the APOL1 gene, G1 and G2, have an increased risk of developing several forms of kidney disease, including focal segmental glomerulosclerosis (FSGS) and HIV-associated nephropathy (HIVAN) [23,24]. APOL1 is a protein component of HDL particles [25] and is believed to have a function in reverse cholesterol transport. Indeed, we previously described cholesterol accumulation in kidneys of transgenic mice with APOL1 risk variants’ expression in association with decreased mRNA expression levels of ABCA1 and ATP binding cassette subfamily G member 1 (ABCG1) [15].

Interestingly, it was found that APOL1 G0 preferably localizes to lipid droplets, while risk variant coded APOL1 translocates to other organelles, including the endoplasmic reticulum (ER). Consequently, it was suggested that ER retention of risk variant coded APOL1 may cause ER stress in podocytes [26]. If and how APOL1 risk variants are associated with kidney diseases through altered lipid metabolism remains to be established.

### 2.3. Disorders Caused by Disrupted Sphingolipids Metabolism

The role of sphingolipids in the pathogenesis of kidney diseases has been comprehensively reviewed [11,27]. Sphingolipid accumulation in glomerular disease of genetic origin was elaborated in detail elsewhere [27]. For example, Fabry disease (FD), a rare inherited disorder of glycosphingolipid metabolism is characterized by a progressive accumulation of globotriaosylceramide in lysosomes of many renal cell types, including podocytes, tubular cells, glomerular endothelial, mesangial and interstitial cells [28]. Renal damage in Fabry disease manifests as proteinuria, hyperfiltration and impaired concentration ability. It is noted that knockout of sphingosine-1-phosphate (S1P) lyase (SGPL1) ortholog in drosophila is associated with defects in nephrocytes and disrupted sphingolipid metabolism. Mutations in this gene have also been identified in humans, exhibited by nephrotic syndrome and other phenotypes such as facultative adrenal insufficiency and immunodeficiency [29].

## 3. Lipid Homeostasis in Podocytes

Lining the epithelial side of the glomerular basement membrane (GBM), podocytes are highly specialized cells with interdigitating foot processes that form the filtration slits [30]. Enriched with cholesterol in the lipid raft-like structure [10,31,32], the slit diaphragm is an essential part of the glomerular filtration barrier [33,34]. Cholesterol synthesis at the ER is catalyzed by HMGCR, a rate-limiting enzyme, whose expression is transcriptionally regulated by sterol regulatory element-binding proteins (SREBPs) [35]. In the event of a cholesterol deficit, cholesterol can be de novo synthesized via HMGCR or cholesterol bound to low-density lipoprotein (LDL) can be delivered into cells via the LDL receptor (LDLR). On the other hand, excess cholesterol can be removed from the cell through ABCA1 and ATP-binding cassette subfamily G member 1 (ABCG1) [10,36]. Cellular cholesterol accumulation activates the cholesterol-esterifying enzyme sterol O-acyltransferase (SOAT1), which then converts free cholesterol to cholesterol esters that are stored in lipid droplets (LD) [37]. Conversely, cholesterol esters can be hydrolyzed to free cholesterol by neutral cholesterol ester hydrolase (NCEH) [10,36]. LDs in different cell types share a similar structure [38,39] consisting of an LD core composed of neutral lipids such as cholesterol esters and triglycerides, which are shielded by a phospholipid monolayer [40]. LDs interact with both the ER and mitochondria where diacylglycerol O-acyltransferase (DGAT), the major enzyme catalyzing triglyceride synthesis, is enriched [41,42]. Composed of a glycerol backbone and three fatty acids, triglycerides are synthesized mainly at the ER from fatty acyl-CoA and diacylglycerol [43]. In podocytes, free fatty acid uptake for triglyceride synthesis occurs via cluster of differentiation 36 (CD36) [10]. On the contrary, fatty acids can also be converted to fatty acyl-CoA and transported into mitochondria for β-oxidation via carnitine palmitoyltransferase 1 (CPT1). The cellular levels of fatty acids, triglycerides and phospholipids are regulated by proliferator-activated receptors (PPARs) [44,45] and SREBPs [46,47]. Therefore, the concept of lowering intrarenal lipid homeostasis can also be achieved by agents targeting the key enzymes or transporters in lipid metabolism.

## 4. Potential Therapeutic Targets

While many enzymes and proteins involved in lipid metabolism may represent drug targets, we will describe below those targets that we believe have attracted a lot of interest in the past few years.

### 4.1. ABCA1/ABCG1

As the major regulator of cholesterol efflux in podocytes, ABCA1 deficiency is associated with podocyte lipid accumulation in glomerular diseases such as diabetic kidney disease (DKD), FSGS and Alport syndrome [48,49,50]. Using a pharmacological ABCA1 inducer in db/db mice, we demonstrated a significant reduction in cholesterol ester content in the kidney cortices in association with improved renal function [48]. The renoprotective effect of the ABCA1 inducer in db/db was associated with a reduction in oxidative stress as indicated by decreased peroxidized cardiolipin species [48]. In another study, we demonstrated that promoting ABCA1-mediated cholesterol efflux similarly prevents renal disease progression in an experimental model of FSGS (adriamycin-induced nephropathy) and of Alport syndrome (Col4a3 KO mice) in association with reduced formation of lipid droplets in glomeruli and reduced accumulation of cholesterol esters in kidney cortices. Mechanistically, we showed that these compounds exercise their effect via binding to oxysterol binding protein-like 7 (OSBPL7), opening the possibility that OSBPL7 could be a novel therapeutic target [49].

We previously demonstrated that treatment of human podocytes with the sera from patients with DKD leads to decreased ABCA1 expression and cholesterol accumulation due to impaired cholesterol efflux and podocyte injury [50,51]. Meanwhile, the removal of cholesterol with methyl-β-cyclodextrin reduced cholesterol accumulation and prevented podocyte apoptosis [51]. In vivo, we demonstrated that cholesterol removal by hydroxypropyl-β-cyclodextrin protects from the progression of renal disease in several mouse models, including mouse models for DKD (ob/ob mice) [51], FSGS (NFATc1nuc) [50] and renal disease associated with Alport Syndrome (Col4a3) [52], though the effect of β-cyclodextrin on cellular cholesterol is ABCA1-independent [53]. In another study, we demonstrated that increased ABCA1 expression and induction of cholesterol efflux by SOAT1 inhibition is necessary to prevent renal injury in both DKD and Alport syndrome [54]. SOAT1 converts free cholesterol to cholesterol esters and maintains the balance of these two lipid species [37]. In support, SOAT1 inhibitor treatment protected podocytes from DKD sera-induced cytotoxicity in vitro and db/db mice from renal injury in vivo through a mechanism dependent on ABCA1-mediated cholesterol efflux. Similarly, SOAT1 inhibition was also found to improve kidney function in Col4a3 KO mice [54].

Exendin-4 is a glucagon-like peptide-1 receptor agonist approved for the treatment of patients with diabetes mellitus. Studies have shown that exendin-4 upregulates ABCA1 expression levels in adipocytes, hepatocytes, and pancreatic cells [55,56,57]. Similar to what was observed in other organs, exendin-4 was also found to decrease renal cholesterol accumulation and to increase cholesterol efflux from glomerular endothelial cells [58]. Injection of exedin-4 in ApoE KO mice fed a high-fat diet (HFD) decreased blood lipid levels and reduced glomerular lipid droplet accumulation. Interestingly, exedin-4 treatment of diabetic apoE^−/−^ mice simultaneously induced glomerular ABCA1 expression [58]. In vitro, human glomerular endothelial cells cultured under high glucose and high cholesterol conditions showed reduced ABCA1 levels, while exendin-4 treatment increased ABCA1 expression and ApoA-I-mediated cholesterol efflux [58].

The above studies suggest that targeting cholesterol efflux represents a new promising therapeutic strategy to ameliorate lipotoxicity-mediated renal injury in renal diseases of metabolic and non-metabolic origin, and that the beneficial effect can be achieved in an ABCA1-dependent or independent manner.

### 4.2. CD36

CD36 is a scavenger receptor protein that enhances cellular fatty acid uptake, a process that is also accompanied by accelerated fatty acid esterification [59]. Increased renal CD36 expression was reported in patients with DKD and was shown to be associated with elevated triglycerides and cholesterol content in the kidney [60,61].

Sulfo-N-succinimidyloleate (SSO) is a specific inhibitor of the fatty acid-binding site on CD36 [62]. Pre-treatment of podocytes with SSO was found to reduce palmitic acid-induced lipid accumulation and cell apoptosis [60]. Other study also proved that SSO decreased palmitate-induced apoptosis in HK2 tubular cells [63], indicating CD36 as a therapeutic target for both glomerular and tubular injury.

In a mouse model of obesity-related glomerulopathy (ORG), increased expression of CD36 in the kidney cortices was found to be associated with nod-like receptor protein 3 (NLRP3) inflammasome activation [64]. In vitro, the same study showed that podocytes in culture stimulated with leptin also exhibited increased expression of CD36 and NLRP3 inflammasome activation, while blocking CD36 with SSO or CD36 siRNA reduced lipid accumulation and podocyte NLRP3 inflammasome activation, suggesting that CD36-mediated lipid uptake plays a pivotal role in inflammasome activation and podocyte injury [64].

Other agents were also reported to be reno-protective via CD36. For example, 5A, an ApoA-I-mimetic peptide that promotes cholesterol efflux, was also found to be an antagonist of CD36 [65]. In a study, where CKD progression in mice after 5/6 nephrectomy (5/6Nx) plus continuous angiotensin II (AngII) infusion was evaluated, CD36 KO or 5A peptide therapy prevented a decline in kidney function, while 5A treatment did not improve renal function in CD36 KO mice, indicating that CD36 is the major target of action [65].

Interestingly, SS-31 a compound that specifically targets the mitochondrial phospholipid cardiolipin [66] was also shown to lower diabetes-induced CD36 expression in db/db mice and to improve high glucose-induced CD36 expression and lipid deposition in HK-2 cells, suggesting that the protective effect of SS-31 on renal function is associated with the suppression of CD36 expression [67].

We recently demonstrated that CD36 interacts with discoidin domain receptor 1 (DDR1) [68], the tyrosine kinase receptor that can be activated by collagens [69]. We found that the interaction of CD36 with DDR1 is associated with increased free fatty acid uptake and contributes to podocyte lipotoxic injury [68]. Moreover, we found that ezetimibe, a cholesterol absorption inhibitor [70], which was shown to exercise a beneficial effect on dyslipidemia associated with CKD [71], inhibits the interaction between CD36 and DDR1, thus suppressing CD36-mediated fatty acid uptake and protecting from renal failure in a mouse model of Alport Syndrome in Col4a3 KO mice [68].

Inhibition of sodium-glucose co-transporter 2 (SGLT2) prevents the reabsorption of filtered glucose, thus leading to a reduction in blood glucose through urinary excretion [72]. Of note, SGLT2 anti-sense oligonucleotide reduced the mRNA levels of CD36 in the hearts of diabetic mice [73]. Additionally, empagliflozin, an SGLT2 inhibitor not only significantly reduced the mRNA and protein levels of CD36 in atrial tissues of Zucker rats, but also lowered the cardiac content of sphingolipids (ceramides and sphingomyelins) and glycerophospholipids [74], indicating a possibly favorable off-target effect. Similar to this study, SGLT2 inhibitors can improve liver fat deposition in type 2 diabetes patients with fatty liver disease [75,76]. These data suggest that the reno-protective effects of SGLT2 inhibitors could potentially be mediated by reducing lipotoxicity in addition to reducing glucose uptake in the proximal tubules.

### 4.3. SREBPs

As an important transcriptional factor that regulates cholesterol and fatty acid synthesis, the overexpression of SREBPs is associated with increased cholesterol and triglyceride content, glomerulosclerosis and proteinuria, which contribute to glomerular diseases, including FSGS [51] and DKD [77,78]. Many SREBP inhibitors have been shown to be reno-protective.

For example, fatostatin is a diarylthiazole derivative that inhibits the activation of SREBPs. Fatostatin blocks the translocation of SREBPs from ER to Golgi through binding to the SREBP cleavage-activating protein (SCAP) [79]. Several studies have investigated the therapeutic potential of fatostatin in mouse models of renal disease. One study demonstrated that fatostatin inhibits renal SREBP activation in the unilateral ureteral obstruction (UUO) model, thereby attenuating inflammatory cell infiltration and apoptosis. Additionally, fatostatin treatment preserved the integrity of the glomerular tubular junction and reduced the formation of atubular glomeruli (glomeruli lose tubular attachment) [80]. Similarly, one study demonstrated that the administration of fatostatin prevented angiotensin II-induced glomerular SREBP-1 elevation, proteinuria, and matrix accumulation in a mouse model of hypertension-induced kidney disease [81]. However, another study by the same group demonstrated that fatostatin only attenuated basement membrane thickening but failed to improve albuminuria, hyperfiltration or kidney fibrosis in a model of type 1 diabetes [82]. Additionally, SREBP inhibition with fatostatin was found to cause kidney injury in nondiabetic mice [82]. These divergent results may be due to the different mouse strains that were used in these studies. Additionally, increased tubular expression of proinflammatory cytokine monocyte chemoattractant protein-1 (MCP-1) was detected after fatostatin treatment [83], indicating that opposite effects should be considered in the development and use of this agent.

A natural product called betulin has also been identified to specifically inhibit the SREBP pathway by promoting the binding of SCAP to insulin-induced genes (INSIGs) and reducing SREBP target gene expression, including the expression of HMGCR and fatty acid synthase (FAS) [84]. In support, betulin was also shown to inhibit the expression of renal SREBPs in db/db mice, thereby reducing renal triglyceride and cholesterol accumulation and preventing proteinuria and podocyte loss [85]. Interestingly, betulin also promotes cholesterol efflux in macrophages by increasing the expression of ABCA1 and ABCG1, which could be an additional mechanism by which betulin reduces intracellular lipid accumulation [86].

Xanthohumol (XN) is another SREBP inhibitor and was shown to ameliorate Western-type diet-induced obesity, diabetes, and fatty liver in mice by repressing the maturation of SREBP-1 [87,88]. Specifically, XN impairs the translocation of the SCAP-SREBP complex and cleavage of SREBPs, thus blocking the maturation of SREBP [88]. Moreover, XN was found to inhibit the secretion and lipidation of apolipoprotein B (ApoB) in HepG2 cells, thus preventing ApoB assembly into a lipoprotein particle in ER membrane and diverting it to the degradation pathway and to decrease triglyceride synthesis by inhibiting DGAT activity [89]. XN treatment of mice on an HFD increased the phosphorylation of AMP-activated protein kinase (AMPK) in the liver and skeletal muscle, while reducing the expression of SREBP-1c, FAS and CD36 [90]. While most studies were performed in other organs, one study investigated the role of XN treatment in the kidney of rats after ischemia/reperfusion-induced kidney injury and demonstrated significantly decreased renal levels of TNF-a and Interleukin 6 (IL-6) when compared to the non-treated group. XN treatment also improved renal function and reduced oxidative stress and lipid peroxidation [91].

At present, of all the SERBP inhibitors discussed above, only XN has progressed to clinical trials and studies of this agent include metabolic syndrome (NCT01367431), oxidative stress (NCT02432651) and Crohn’s disease (NCT04590508). Considering the effect of XN on systemic and renal lipid metabolism, SREBP inhibitors may prove promising for the treatment of patients with renal disease.

### 4.4. PPARs

As nuclear receptor proteins, PPARs heterodimerize with retinoid X receptor (RXR), thus stimulating the transcription of genes involved in fatty acid β-oxidation and lipolysis [92]. The role of PPARs in kidney diseases has been comprehensively reviewed elsewhere [93,94,95]. Notably, genetic analysis has shown reduced expression of PPARα and PPARγ in kidneys of patients with DKD [61], suggesting that PPARs could be a potential drug target for the treatment of DKD.

Fibrates are synthetic PPARα ligands. Studies have shown that fibrates, such as fenofibrate, can alleviate albuminuria and glomerular lesions in HFD-fed mice by attenuating triglyceride accumulation in the kidney. Fenofibrate also reduces the glomerular expression of 4-hydroxynonenal, a lipid peroxidation marker, while the expression of lipolytic genes, such as acyl-CoA oxidase (ACO) and CPT-1, was found to be increased in the kidney cortex [96]. Others demonstrated that fenofibrate reduces renal lipotoxicity by inhibiting the accumulation of intra-renal free fatty acids and triglycerides in db/db mice, whereby its action seems to be associated with activation of the AMPK-PPAR-γ coactivator-1α (PGC-1α) pathway [97]. Similarly, feeding db/db mice with a diet containing fenofibrate improves kidney function, in addition to improved diabetes [98]. In another study, fenofibrate treatment was shown to protect podocytes from doxorubicin-induced apoptosis in vitro, and it also attenuated doxorubicin-induced proteinuria and podocyte foot effacement in vivo [99].

Insulin sensitizers, thiazolidinediones (TZDs), are synthetic ligands of PPARγ. The activation of PPARγ by troglitazone, an agent of the TZD family, inhibits the development of albuminuria and glomerular extracellular matrix in streptozotocin-injected mice [100]. Similarly, troglitazone prevents glomerular dysfunction in streptozotocin-induced diabetic rats by inhibiting the diacylglycerol-protein kinase C pathway [101]. It has been noted that troglitazone induces CD36 expression in wild-type but not in PPAR-γ deficient macrophages, indicating that CD36 is a target gene for PPAR-γ in macrophages [102]. Additionally, another TZD drug pioglitazone improved renal function of Otsuka Long-Evans Tokushima fatty (OLETF) rats, an experimental model of diabetic nephropathy. Pioglitazone ameliorated podocyte hypertrophy by inhibiting de novo protein synthesis. These data suggest podocytes are important targets of TZDs administration in DKD [103].

Several PPARα and PPARγ agonists are FDA-approved. For example, fenofibrate, a PPARα agonist that is currently utilized for the treatment of hypertriglyceridemia, is currently being evaluated in a phase 3 trial for the treatment of patients with diabetic kidney disease (NCT03869931). Additionally, a phase 4 trial for the treatment of patients with type II diabetes and dyslipidemia has been completed (NCT02153879). The PPARγ agonist pioglitazone is undergoing a phase 4 trial in patients with CKD (NCT03471117) and troglitazone completed a phase 3 trial in patients with diabetes (NCT00116545).

Besides these two classes of agents, agents such as Wy14643 and prostaglandin J2 were used in mice to activate PPARα and PPARγ, respectively. These compounds were able to increase liver X receptor (LXR) α and ABCA1 gene expression and enhanced ApoA1-mediated cholesterol efflux, thereby preventing intracellular lipid accumulation in mesangial cells [104]. Additionally, Wy14643 was found to prevent the inhibition of fatty acid oxidation in proximal tubular cells during acute renal failure [105], suggesting PPAR agonists may lower renal lipids by lowering toxic fatty acid accumulation.

### 4.5. FXR

The farnesoid X receptor (FXR) is a ligand-activated nuclear receptor that regulates cholesterol and fatty acid metabolism [106,107]. FXR forms a heterodimer with RXR, thereby mediating the conversion of cholesterol to bile acids [108]. It was demonstrated that activation of FXR reduces lipogenesis by inhibiting SREBP-1c and FAS expression [108]. FXR also induces the activation of PPAR-α in human liver cells [109]. Several FXR agonists have been shown to exercise renoprotective effects. 

The FXR agonist, obeticholic acid attenuated kidney injuries such as glomerular matrix expansion and interstitial fibrosis in uninephrectomized obese mice. FXR activation is associated with reduced lipids in the remaining kidney. The renal expression of acyl-CoA oxidase 1 (ACOX1), CPT1a and PPARα were elevated, indicating more renal lipolysis and fatty acid oxidation [110].

In support of a beneficial effect of FXR agonists in the treatment of kidney diseases, streptozotocin injection in FXR KO caused an accelerated progression of DKD with glomerular foam cell accumulation and mesangial matrix expansion. Similarly, treatment with the FXR agonist INT-747, an obeticholic acid, was shown to protect from renal injury as manifested by decreased proteinuria, glomerulosclerosis and tubulointerstitial fibrosis, as well as reduced renal SREBPs expression and lipid accumulation [111]. Similarly, FXR activation by INT-747 prevented renal triglyceride accumulation in mice fed on a Western diet. In the kidney, genes relevant to fatty acid oxidation and lipid catabolism, such as PPAR-α, PGC-1α and CPT1a, were upregulated, while renal expression of SREBP, acetyl-CoA carboxylase (ACC) and FAS were downregulated [112]. Like FXR, Takeda G protein-coupled receptor 5 (TGR5) is another bile acid-activated nuclear receptor. Treatment of db/db mice with INT-747 or with the dual FXR/TGR5 agonist INT-767 reduces urinary albumin excretion, and both regulate the lipogenesis pathway mediated by SREBP-1 [113]. Interestingly, renal FXR and TGR5 expression are decreased with aging, and the dual FXR/TGR5 agonist INT-767 can also reverse the development of age-related albuminuria in mice [114]. INT-747 is currently being tested in phase 4 clinical trial for the treatment of patients with liver cirrhosis (NCT02308111) and a phase 2 clinical trial for the treatment of patients with diabetes has been completed (NCT00501592).

GW4064 is another FXR agonist that has been studied in vitro and in vivo. In a rat model of maternal obesity, which predisposes the offspring to glucose intolerance, renal FXR expression was found decreased, accompanied by the renal expression of pro-inflammatory and fibrotic factors. The same change was observed in HK2 cells exposed to high glucose, which was reversed by GW4064 [115]. Interestingly, renal mesangial cells in the presence of GW4064 show inhibition of the expression of SREBP-1c and other lipogenic genes as well as inflammatory genes [116].

### 4.6. LXR

Like FXRs, LXRs are also nuclear receptors that act as lipid sensors for cholesterol and bile acids. Normal LXR function is a necessity to prevent renal lipid accumulation, which was found to be associated with glomerular injury [117]. In support of a crucial role of LXR in kidney function, LXR KO mice exhibited increased albuminuria and glomerular lipid accumulation, which was further accelerated in a diabetic environment [118]. It is noted that the induction of ABCA1 and ABCG1 expression are the best-characterized effects of LXR activation [108].

LXR activation using the synthetic agonist GW3965 in LDLR KO mice fed with a Western diet and/or injected with streptozotocin to induce hyperlipidemia/hyperglycemia-induced renal lesion, was shown to increase renal expression of ABCA and ABCG1, and to reduce lipid accumulation and lipid droplet formation, and improve renal function [119]. Similarly, the LXR agonist *N*,*N*-dimethyl-hydroxycholenamide (DMHCA) was shown to attenuate albuminuria and glomerular injury in LXR KO mice, which was indicated by less mesangial expansion and foam cell by periodic acid–Schiff (PAS) staining [118]. Treatment of DMHCA also reduced inflammatory marker expression and renal lipid accumulation. Similarly, the administration of another LXR agonist T0901317 to mice with streptozotocin-induced diabetes not only decreased urinary albumin excretion, but also ameliorated glomerular hypertrophy, mesangial matrix expansion, and macrophage infiltration by inhibiting osteopontin expression [120]. At present, only LXR agonists such as RGX-104 are undergoing a phase 1 trial in patients with malignant neoplasms (NCT02922764), and VTP-38543 completed a phase 2 trial in patients with atopic dermatitis (NCT02655679), but several studies have shown that LXR agonists cause adverse events which hinder their progression [121].

### 4.7. APOL1

Although to date, there is no evidence suggesting that any particular therapeutic approach in the treatment of APOL1 nephropathy would be superior to the standard treatments [122], APOL1 allele-targeting approaches are currently being explored. For example, targeting APOL1 mRNA using an antisense oligonucleotide (ASO) was shown to be an effective approach to protect APOL1 G1 transgenic mice from IFN-γ–induced proteinuria [123]. In a recent study, we demonstrated that human urinary podocytes expressing APOL1 risk variants are characterized by lipid accumulation and mitochondrial dysfunction while treatment with an ABCA1 inducer or LXR agonist ameliorated lipid-mediated mitochondrial dysfunction [124], suggesting that inducers of reverse cholesterol transport may represent a new therapeutic targeting strategy for patients with APOL1-associated kidney disease. Currently, two APOL1 inhibitors, i.e., VX-147 [125] and the antisense oligonucleotide AZD2373 are being tested in Phase 2 (NCT04340362) and Phase 1 (NCT04269031) clinical trials, respectively, for the treatment of patients with APOL1-associated kidney disease. However, it is still debatable if APOL1-associated kidney disease is due to loss-of-function or gain-of-function [126].

### 4.8. PCSK9 

Reduced lipoprotein lipase activity but increased low-density lipoprotein (LDL) production and pro-protein convertase subtilisin/kexin type 9 (PCSK9) expression have been associated with CKD-related dyslipidemia [127]. PCSK9 facilitates the degradation of LDLR in the liver, thus leading to reduced LDL clearance [127]. Interestingly, mice injected with nephrotoxic serum and mice with selective podocyte ablation were found to have increased plasma PCSK9 levels in association with increased proteinuria, hypertriglyceridemia and hypercholesterolemia. On the other hand, knockout of PCSK9 was found to ameliorate dyslipidemia after treatment with nephrotoxic serum [128]. In an earlier study, Pcsk9^−/−^ mice were found to have decreased lipid accumulation in hepatocytes, which exhibited resistance to liver steatosis [129]. Evolocumab and alirocumab are two monoclonal antibodies and PCSK9 inhibitors that effectively reduce systemic LDL levels along with maximum-tolerated statin therapy in patients with normal to moderately impaired kidney function [130]. Additional targets of PCSK9 include CD36 and Niemann-Pick C1-like 1, suggesting that PCSK9 inhibitors may regulate lipid metabolism in an LDLR-independent way [131]. Evolocumab is now being tested in Phase 4 in patients with atherosclerosis, type 2 diabetes, and microvascular dysfunction (NCT03900026, NCT03829046).

### 4.9. CETP

Cholesteryl ester transfer protein (CETP) regulates the distribution of cholesterol esters and triglycerides between lipoproteins. Increased serum CETP levels lead to depletion of HDL cholesterol [132], which can be observed in nephrotic patients [133]. Meanwhile, CETP activity was found to correlate with HDL-cholesterol levels in patients with stage V CKD, although neither was found to be associated with increased cardiovascular events [134]. It has been demonstrated that circulating CETP can increase free cholesterol accumulation in the islets of transgenic mice [135], indicating CETP inhibition could potentially decrease tissue cholesterol accumulation, in addition to its effect on serum levels. Currently, evacetrapib is the only CETP inhibitor that was found to also reduce major coronary events. It was recently demonstrated that anacetrapib increases cholesterol efflux capacity independent of changes in HDL but this may be dependent on gender (male) and haptoglobin genotype (1-1 homozygotes) [136], indicating that determining the haptoglobin genotype of patients could be beneficial in the use of CETP inhibitors.

### 4.10. LDL

LDL apheresis was first used in the treatment of familial hypercholesterolemia [137]. LDL apheresis has been implemented in the treatment of patients with nephrotic syndrome, specifically FSGS [138], which often present with increased serum LDL levels. For instance, the LDL-apheresis device Liposorber LA-15^®^ System is recognized as an alternative therapy for patients with FSGS and is furthermore under investigation for its safety and efficacy in drug-resistant pediatric primary FSGS. A phase 3 trial that compares the PCSK9 inhibitor evolocumab to LDL apheresis in patients with hypercholesterolemia has been completed (NCT03429998). If and how LDL-apheresis mediated a reduction in proteinuria in NS is directly linked to the removal of LDL or of other circulating factors remains to be established.

## 5. Conclusions

**Figure 1:**
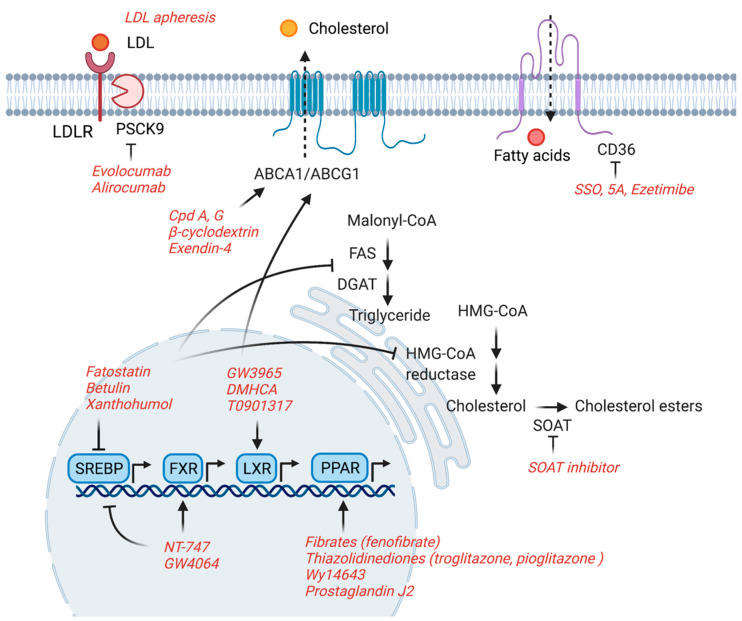
Potential therapeutic targets of lipid-modifying agents. ABCA1/ABCG1: ATP binding cassette subfamily A/G member 1; CD36: cluster of differentiation 36; DGAT: diacylglycerol O-acyltransferase; FAS: fatty acid synthase; FXR: farnesoid X receptor; HMGCR: 3-hydroxy-3-methyl-glutaryl-CoA reductase; LDL: low-density lipoprotein; LDLR: LDL receptor; LXR: liver X receptor; PCSK9: pro-protein convertase subtilisin/kexin type 9; PPAR: proliferator-activated receptor; SOAT: sterol O-acyltransferase; SREBP: sterol regulatory element-binding protein.

**Figure 2:**
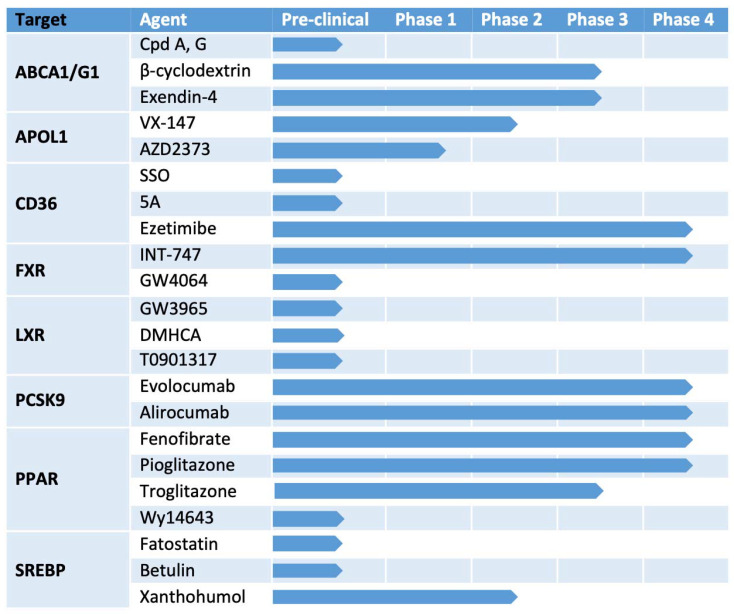
The status of different drugs under development that target lipid metabolism. ABCA1/ABCG1: ATP binding cassette subfamily A/G member 1; APOL1: Apolipoprotein L1; CD36: cluster of differentiation 36; FXR: farnesoid X receptor; LXR: liver X receptor; PCSK9: pro-protein convertase subtilisin/kexin type 9; PPAR: proliferator-activated receptor; SREBP: sterol regulatory element-binding protein.

Modifying intracellular lipids provides new therapeutic opportunities for the treatment of patients with glomerular diseases. In this review, we have summarized the current evidence obtained from in vitro and in vivo studies, which suggests that lipid-modifying agents can be used to attenuate renal cell or kidney injury, respectively. Key enzymes and transporters involved in the regulation of lipid metabolism are often intertwined with each other in the progression of CKD and serve as druggable targets (Figure 1). While some of the mentioned agents are still in the pre-clinical stage of investigation, others have progressed to clinical studies (Figure 2). Though LDL-apheresis has gained a lot of interest in the past few years, the development of less invasive pharmacological strategies acting on specific targets will likely offer novel treatment strategies for glomerular disorders of both metabolic and non-metabolic origin.
